# Cardiovascular Implications of Epilepsy: Unraveling the Elevated Risk of Mortality

**DOI:** 10.7759/cureus.59921

**Published:** 2024-05-08

**Authors:** Sunaina Addanki, Andrew Schleffer, Rishabh Kasarla, Stephen Ely

**Affiliations:** 1 Medical School, Nova Southeastern University Dr. Kiran C. Patel College of Allopathic Medicine, Fort Lauderdale, USA; 2 Cardiothoracic Surgery, Nova Southeastern University Dr. Kiran C. Patel College of Allopathic Medicine, Fort Lauderdale, USA

**Keywords:** cardiac death in epilepsy, sudden unexpected death in epilepsy, epilepsy, cardiac-related death, sudep

## Abstract

Introduction

Epilepsy is a complex prevalent seizure disorder impacting a significant number of individuals worldwide. Those with epilepsy face the possibility of experiencing sudden unexpected death in epilepsy (SUDEP). When examining the relationship between epilepsy and SUDEP, cardiac-related deaths (CRD) may be considered a driving force. We hypothesize that patients with epilepsy are at higher risk of CRD than those without epilepsy. While utilizing the National Institutes of Health (NIH) All of Us Researcher Program (AoU) database, we also explored the relationship between epilepsy and cardiac-related deaths and propose potential connective mechanisms between the two conditions.

Methods

Baseline data from the National Institutes of Health All of Us Researcher Program was used to evaluate the relationship between cardiac-related deaths and epilepsy. A retrospective cohort study was conducted where individuals with epilepsy and without epilepsy were matched by inclusion and exclusion criteria including death, cardiac-related death, and epilepsy. Additionally, the prevalence of cardiac-related deaths was compared to neurological, respiratory, and hepatic-related deaths for patients with epilepsy to identify emerging causes of SUDEP.

Results

Among patients with a history of epilepsy, the prevalence of CRD was 45 (17.3%) compared to 305 (11%) in the control group. This difference was statistically significant by p<0.0042 with an odds ratio (OR)=1.698, 95%CI 1.214-2.379. Additionally, there was the highest number of significant cardiac-related deaths amongst patients with epilepsy compared to patients without epilepsy as opposed to different mechanisms of death such as acute respiratory failure, acute hepatic failure, and hypoxic brain injury.

Conclusion

This study indicates that epileptic patients have a statistically significant higher prevalence of cardiac-related deaths. Additionally, cardiac-related deaths constitute a significantly higher proportion of fatalities amongst patients with epilepsy compared to other causes of SUDEP. Potential mechanisms for these findings may include seizure-induced arrhythmias, hypoxia-induced cardiac arrest, autonomic dysregulation, and neurotransmitter disequilibrium. The results of our study suggest promising directions for future research in identifying predictors of cardiac-related deaths with proposed cardiac monitoring protocols as preventative strategies for epileptic patients in efforts to reduce the prevalence of SUDEP.

## Introduction

Epilepsy is recognized as the most common seizure disorder affecting over 50 million individuals worldwide. Approximately one in 26 people will develop epilepsy throughout their lifetime [1,2]. Patients with epilepsy are known to have a higher risk of sudden unexpected death, commonly referred to as sudden unexpected death in epilepsy (SUDEP) [3,4]. 1.16 cases of SUDEP occur for every 1,000 people with epilepsy [2]. Most cases of SUDEP have been identified during or immediately after a seizure [5]. The exclusion/absence of trauma, drowning, toxicology or other anatomical factors contributing to a patient's death is required for the classification of SUDEP [4]. SUDEP is known to encompass deaths relating to cardiac, neurological, respiratory, and hepatic conditions or complications. From a cardiac standpoint, complications such as cardiac arrest, coronary artery disease, ventricular fibrillation, and electromechanical dissociation may prevail. Neurologically, hypoxic brain damage and hypoxic ischemic encephalopathy have been identified as possible mechanisms of SUDEP. When considering respiratory complications, conditions such as neurogenic pulmonary edema, hypoventilation, central apnea, and obstructive respiratory failure are possible explanations for SUDEP. Finally, liver pathologies may also contribute to SUDEP which generally arises from acute hepatic failure. When examining the relationship between epilepsy and SUDEP, cardiac-related deaths (CRD) may be considered a driving force.

Multiple connections between SUDEP and cardiac-related deaths have been proposed, however, the mechanism of SUDEP is not well understood [6]. A combination of seizure-induced arrhythmias, genetic anomalies, autonomic disturbances, and neurotransmitter imbalances may collectively play a role in the increased risk of cardiac-related death observed in epileptic patients [6,7]. Our aim is to test the hypothesis that there is a higher risk of CRD in patients with epilepsy compared to those without epilepsy while proposing potential connective mechanisms between CRD and epilepsy.

## Materials and methods

Baseline data from the National Institutes of Health (NIH) All of Us Researcher Program (AoU), was used to evaluate the relationship between cardiac-related deaths and epilepsy. This AoU workbench is a cloud-based platform where approved researchers can access and analyze data. Systemized Nomenclature of Medicine (SNOMED) codes and electronic health record (EHR) measurements were used to identify this data. Results are in compliance with the AoU Data and Statistics Dissemination Policy prohibiting disclosure of groups under 20 patients. AoU enrollment started in May 2018 and enrolled participants 18 years of age or older from a network with over 340 recruitment sites. The researcher workbench includes over 727,00 participants. The consented data includes surveys, EHR data, and physical measurements such as systolic and diastolic blood pressure, height, weight, heart rate, waist and hip measurement, wheelchair use, and current pregnancy status. Participant privacy was protected with a series of data transformations. The Workbench’s tools were used for selecting groups of participants (Cohort Builder), creating datasets for analysis (Dataset Builder), and Workspaces with Jupyter Notebooks (Notebooks) to analyze data. The notebooks enable use of saved datasets and direct query using R and Python 3 programming languages. We used R version 4.0.3 (R Foundation for Statistical Computing, Vienna, Austria) to perform the analyses. We used Microsoft Excel (Redmond, WA, USA) to create figures to display the cardiac related death prevalence and 95% confidence intervals.

A retrospective cohort study was conducted where individuals with epilepsy and without epilepsy were matched by inclusion and exclusion criteria including death, cardiac-related death, and epilepsy. Four different groups were identified by selecting for and excluding certain criteria. The four groups included participants matched by health surveys and were grouped by the following criteria: Group 1: included epilepsy, included cardiac-related death, Group 2: included epilepsy, excluded cardiac-related death, Group 3: excluded epilepsy, included cardiac-related death, and Group 4: excluded epilepsy, excluded cardiac-related death. The SNOMED codes for epilepsy included location-related epilepsy, generalized epilepsy, location-related (focal) (partial) idiopathic epilepsy, refractory epilepsy, tonic-clonic epilepsy, generalized convulsive epilepsy, refractory localization-related epilepsy, idiopathic generalized epilepsy, epilepsy-not refractory, refractory generalized convulsive epilepsy, refractory idiopathic generalized epilepsy, status epilepticus due to generalized idiopathic epilepsy, localization-related epilepsy, cryptogenic generalized epilepsy, partial-epilepsy with impairment of consciousness, status epilepticus, refractory generalized nonconclusive epilepsy, temporal lobe epilepsy, reflex epilepsy, frontal lobe epilepsy, menstrual epilepsy, localization-related cryptogenic epilepsy, occipital lobe epilepsy, partial occipital epilepsy, and post-traumatic epilepsy. The SNOMED codes for cardiac-related death included cardiac arrest, ventricular fibrillation, cardiac arrest due to cardiac disorder, and electromechanical dissociation. Individuals with epilepsy who died of a cardiac-related death were labeled the experimental group while the individuals without epilepsy who died of a cardiac-related death were labeled the control group.

Additionally, the prevalence of cardiac-related deaths was compared to neurological, respiratory, and hepatic-related deaths for patients with epilepsy. Groups of patients were selected for using inclusion and exclusion criteria. SNOMED codes for neurological deaths consisted of brain damage due to hypoxia and hypoxic ischemic encephalopathy. SNOMED codes for respiratory failure consist of neurogenic pulmonary edema and hypoventilation. Finally, SNOMED codes for hepatic failure consisted of acute hepatic failure, and fulminant hepatic failure. Chi square analysis was run to identify significance and relative risk using the Prism Statistical software (GraphPad, San Diego, CA, USA).

## Results

**Figure 1:**
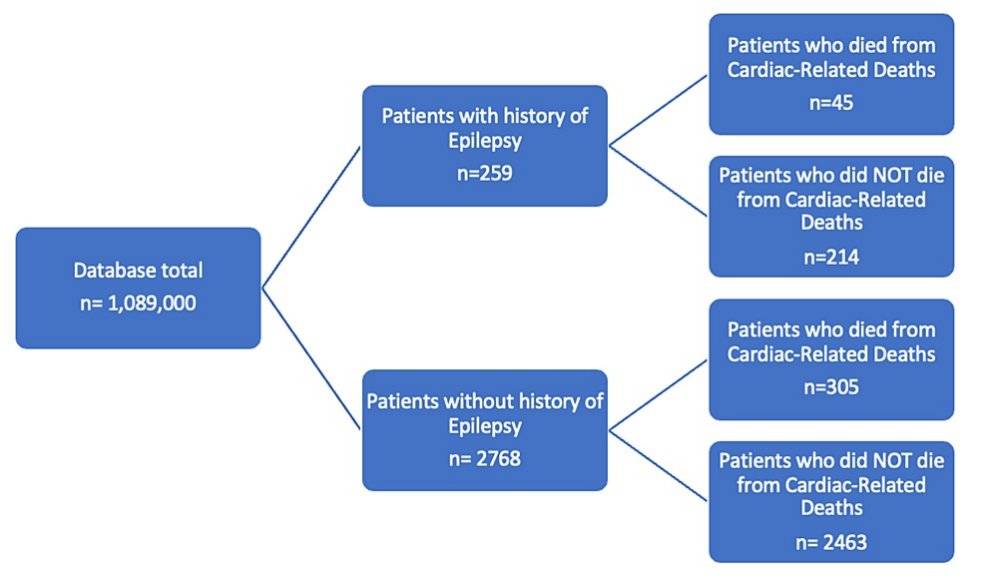
Diagram depicting grouping of patients matched by inclusion and exclusion criteria which included health surveys, history of epilepsy, and cardiac-related deaths.

Using the All of Us research database, 1,089,000 participants were matched for the deceased population group with conditions including epilepsy, and cardiac-related deaths. The experimental group yielded 45 patients while the control group yielded 305 patients (Figure 1).

**Figure 2:**
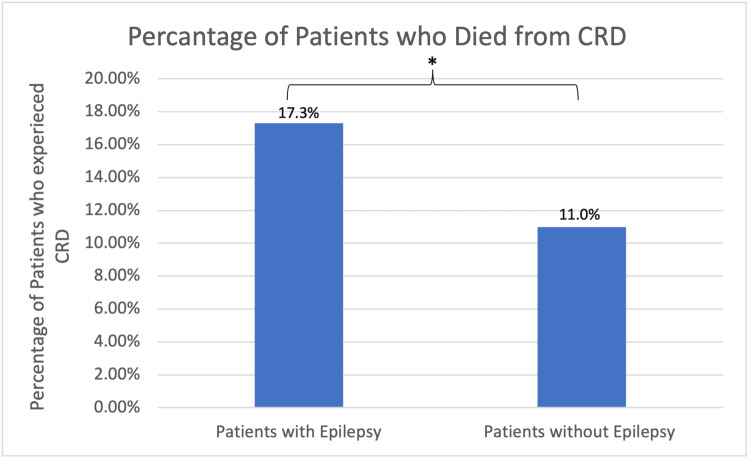
The prevalence of cardiac-related death was (17.3%) in the epilepsy group compared to (11.0%) in the control group. This difference was statistically significant by p<0.0042 with an OR=1.698, 95%CI 1.214-2.379. CRD: cardiac-related deaths, OR: odds ratio

Among patients with a history of epilepsy, the prevalence of CRD was 45/259 (17.3%) compared to 305/2768 (11.0%) in the control group (Figure 2). This difference was statistically significant by p<0.0042 with an odds ratio (OR)=1.698, 95%CI 1.214-2.379 (Figure 2).

**Figure 3:**
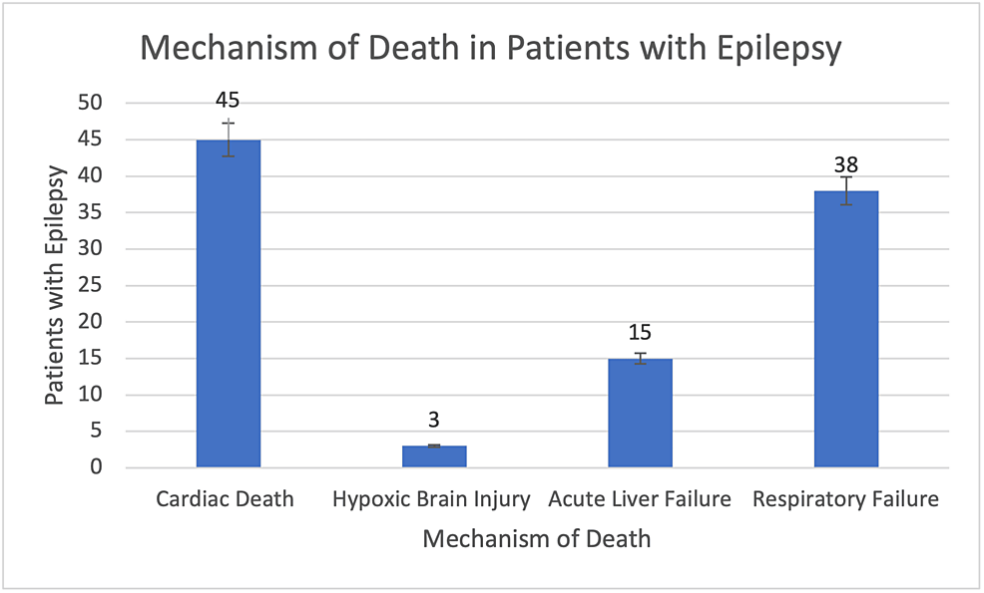
The number of epilepsy patients who died of multiple different mechanisms including cardiac-related deaths, hypoxic brain injuries, acute liver failure, and acute respiratory distress. The number of patients with epilepsy who died of cardiac-related deaths were statistically significant compared to patients without epilepsy with a p-value <0.0042. However, deaths related to hypoxic brain injuries, acute liver failure, and acute respiratory failure were statistically insignificant with p-values less than 0.08, 0.12, and 0.28 respectively.

Cardiac-related deaths were compared to acute respiratory failure, acute hepatic failure, and hypoxic brain injury to determine if cardiac-related deaths played the largest role in SUDEP. Cardiac-related deaths emerged as the highest number of deaths amongst individuals with epilepsy with 45 patients (Figure 3). Amongst the four possible mechanisms of injury for SUDEP, cardiac-related deaths were the only statistically significant group that contributed to the greatest number of deaths. Deaths related to hypoxic brain injuries, acute liver failure, and acute respiratory failure were statistically insignificant with p-values less than 0.08, 0.12, and 0.28 respectively. 

**Figure 4:**
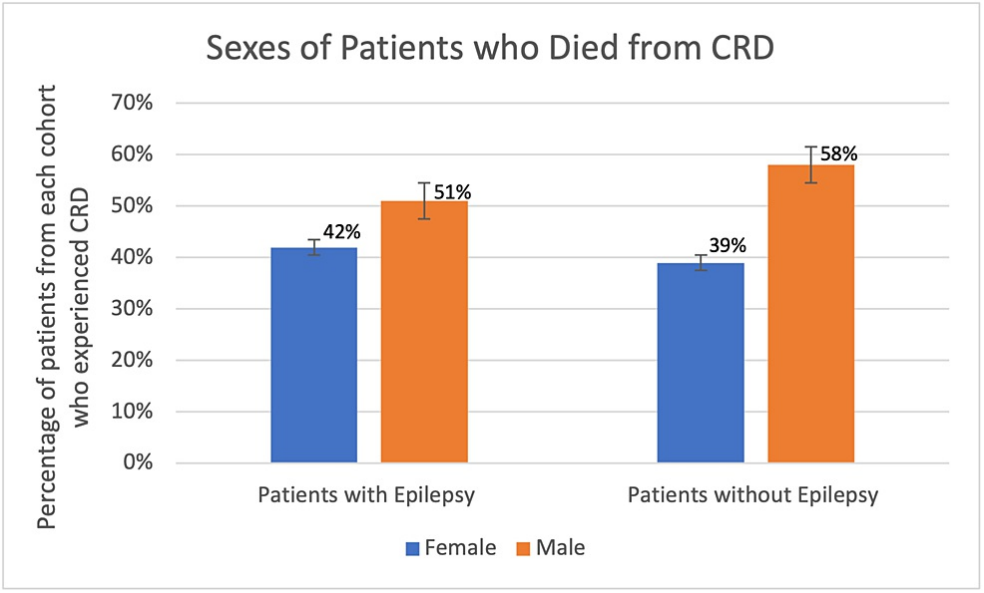
Percentages of patients who died of CRD with and without history of epilepsy for each sex. There were no significant differences in CRD in patients with or without epilepsy as related to sex. Patients who were not accounted for answered as non-disclosed. CRD: cardiac-related death

**Figure 5:**
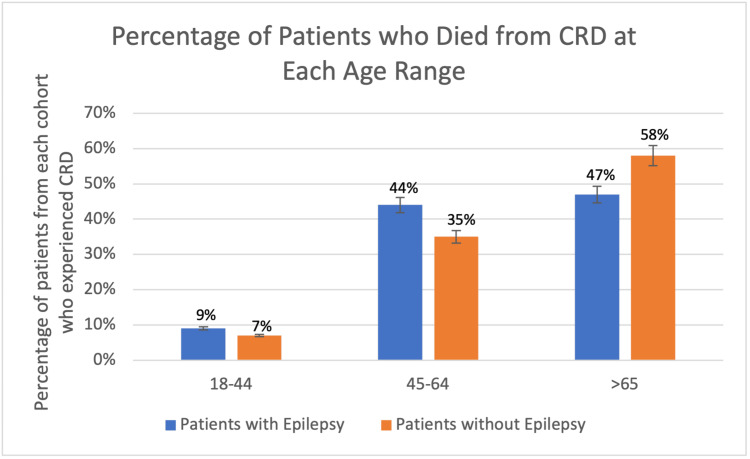
Percentage of patients who died of CRD with and without history of epilepsy at each age range. CRD: cardiac-related death

From a demographic standpoint, overall males accounted for a greater proportion of patients with epilepsy with and without cardiac-related deaths as 51% and 48% of the population respectively (Figure 4). When considering the age ranges, in both the experimental and control group, the largest percentage of patients with cardiac-related deaths was greater in the age >65 with epilepsy group with 58% (176 patients) and 47% (21 patients) in the group without epilepsy, respectively (Figure 5).

## Discussion

Our primary aim is to explore the relationship between epilepsy and cardiac-related deaths using patient data from the NIH All of Us Researcher’s Program. Based on our results, we hypothesize that cardiac-related deaths may be considered a driving force in sudden unexpected death in epilepsy. Our results indicate that the prevalence of cardiac-related death was statistically greater in the epilepsy group compared to the control group without epilepsy. In addition, the percentage of patients who died of cardiac-related death was statistically greater than the percentage of patients who died of neurological, hepatic, and respiratory-related deaths. These results support our hypothesis by demonstrating that not only does epilepsy lead to a greater incidence of cardiac-related deaths, but, amongst various causes of SUDEP in epileptic patients, cardiac-related deaths constitute a significantly higher proportion of fatalities compared to most other causes.

Our secondary aim is to propose potential connective mechanisms between epilepsy and cardiac-related deaths. The two complex conditions share a notable connection that has become a focus of recent research. The literature demonstrates a multifaceted linkage encompassing seizure-induced arrhythmias, autonomic dysregulation, and neurotransmitter disequilibrium. Seizure-induced arrhythmias can emerge from a cascade of physiological alterations including catecholamine release, blood pH changes, and electrolyte shifts [4]. Genetic mutations, notably in SCN1A, a voltage-gated sodium channel, and KCNA1, a voltage-gated potassium channel, are recognized as predisposing factors that can facilitate susceptibility to both seizures and arrhythmias. This is due to the presence of SCN1A channels in the cerebral and cardiac nodal structures, and KCNA1 in the brain and vagus nerve, which underscores the dualistic impact of the ion channelopathies [3,8]. These genotypic variations not only enhance seizure susceptibility but also contribute to autonomic system instability [8]. 

Additionally, seizures have been correlated to autonomic dysfunction in several studies, possibly leading to cardiac-related deaths. Mutations of the SCN1A and KCNA1 genes have been shown to lead to autonomic destabilization [3]. This destabilization leads to reduction in heart rates and altered baroreflex sensitivities. This eventually may lead to hypoperfusion of organs, secondary effects on respiration and eventually cardiac arrest in severe cases [4]. In addition, seizure-induced hormonal and metabolic changes may contribute to SUDEP when combined with dysregulation in cardiac and respiratory physiology as this impairs the human body’s ability to cope with the stress of the seizures [5]. 

Seizure-induced hormonal and metabolic shifts may contribute to SUDEP when in conjunction with dysregulation in cardiac and respiratory physiology [4]. Moreover, the disruption of the neurotransmitter homeostasis during seizures, particularly the release of opioids leading to reduced breathing and post-ictal apnea, can possibly lead to hypoxemia and acidosis leading to failure of recovery of cortical function and eventual cardiac failure, increasing the risk of SUDEP [9,10]. In addition, research shows that serotonergic neurons in the medullary raphe have reduced activity during and after seizures, which can negatively impact breathing, heart function, and alertness, which may also lead to cardiac-related death [11,12]. 

While our study addresses a unique topic that hasn’t been explored heavily, our study is subject to certain limitations. The results of our study are derived from a large database featuring a compilation of patient electronic health records and laboratory results. Despite the size and diversity of the NIH AoU database there are some limitations that must be addressed. As EHRs were limited to single healthcare networks, out-of-network care individuals were not captured in this population. We currently do not have information on data completeness from each recruitment site in the AoU Research Program. Furthermore, the geographic representation of the AoU research program places more emphasis on regions with healthcare provider organizations that have larger recruitment funding. This investigation employed a retrospective cohort methodology which limits the ability to pinpoint confounding factors in pathogenesis.

Having established that cardiac-related deaths may be considered a driving force of SUDEP, there are several possible avenues for future studies. One such study could identify patterns and potential predictors of cardiac-related deaths in SUDEP using long-term data on general cardiac health such as EKGs, echocardiograms, as well as data collected from cardiac monitoring techniques during epileptic seizures that observe any immediate cardiac changes. Analyzing the changes in cardiac measurements in the long-term and immediate pre-ictal periods can allow physicians to intervene early and prevent SUDEP.

Another future direction for our study could be to create and implement cardiac monitoring protocols for individuals with epilepsy that have high risk factors of death to prevent SUDEP. This could include utilization of special devices, frequent check-ups with a cardiologist, genetic testing for ion channelopathies, prioritizing autonomic functional testing, examining hormonal and metabolic health status, and also neurotransmitter testing.

## Conclusions

Our study supports the hypothesis that cardiac-related deaths play a significant role in SUDEP amongst individuals with epilepsy. With a statistically higher prevalence of cardiac-related deaths in the epilepsy group compared to the control group, the importance of cardiac events as a primary contributor to SUDEP is being emphasized. Proposed mechanisms for this include the complex interplay between seizures and arrhythmias, autonomic dysregulation, and neurotransmitter disequilibrium. While our study is subject to limitations, it suggests promising directions for future research in identifying predictors of cardiac-related deaths with proposed cardiac monitoring protocols as preventative strategies for epileptic patients in efforts to reduce the prevalence of SUDEP. 
